# Ozonated olive oil inhibits melanoma proliferation by inducing ferroptosis

**DOI:** 10.1016/j.bbrep.2025.102267

**Published:** 2025-09-17

**Authors:** Seong-Jin An, Jun-Ichi Kashiwakura, Akira Katsuyama, Sumihito Togi, Yuichi Kitai, Ryuta Muromoto, Toshiaki Miura, Satoshi Ichikawa, Tadashi Matsuda

**Affiliations:** a Department of Immunology, Graduate School of Pharmaceutical Sciences, Hokkaido University, Sapporo, Hokkaido, Japan; b Department of Life Science, Faculty of Pharmaceutical Sciences, Hokkaido University of Science, Sapporo, Hokkaido, Japan; c Laboratory of Medicinal Chemistry, Center for Research and Education of Drug Discovery, Hokkaido University, Sapporo, Hokkaido, Japan; d Center for Clinical Genomics, Kanazawa Medical University Hospital, Ishikawa, Kanazawa, Japan

**Keywords:** Melanoma, Ozonated olive oil, Ferroptosis, Lipid peroxidation

## Abstract

Although ozone is a potent oxidant that can damage lungs and skin after prolonged exposure, ozonated olive oil (OZO) exhibits antimicrobial, anti-inflammatory, and wound-healing effects. Here, we describe a novel application of OZO in melanoma therapy. Treatment with OZO markedly inhibited the proliferation of both human and murine melanoma cells, while sparing normal human keratinocyte. At the molecular level, OZO upregulated ferroptosis-related genes, decreased intracellular glutathione (GSH) and GPX4 protein levels and accelerated lipid peroxidation. Critically, OZO-induced growth inhibition in melanoma cells was prevented by ferroptosis inhibitors (ferrostatin-1 and deferiprone), but not by inhibitors of apoptosis or necroptosis. Taken together, these findings offer new therapeutics strategy for treating melanoma by inducing ferroptosis.

## Highlights

• Ozonated olive oil (OZO) significantly inhibits the growth of human and murine melanoma cells.
• OZO induces ferroptosis-related genes, including HMOX1 and SLC7A11, and lipid peroxidation.
• OZO reduces intracellular GSH and GPX4 protein levels.
• Ferroptosis inhibitors rescue melanoma cells from OZO-induced growth inhibition.

## 1 Introduction

Malignant melanoma, an aggressive malignancy arising from the transformation of epidermal melanoma arises from melanocytes by accumulated genetic alterations, poses a formidable clinical challenge due to its rapid progression to metastatic disease [1,2]. While surgical excision is effective for localized tumours, the management of advanced melanoma has been revolutionized by targeted therapies, such as BRAF-MEK inhibitors, and by immune checkpoint inhibitors, which have significantly improved patient survival [[2], [3], [4]]. Nevertheless, the prognosis for patients with metastatic disease remains guarded. The long-term efficacy of this advanced treatment is frequently undermined by the near-inevitable development of intrinsic or acquired therapeutic resistance, leading to treatment failure [1,2]. This clinical impasse highlights an urgent need for novel strategies that can overcome these resistance mechanisms by engaging alternative cell death pathway.

Ferroptosis, a form of regulated cell death characterized by iron-dependent lipid peroxidation, is mechanistically distinct from other cell death pathways like apoptosis, autophagy, pyroptosis, and necroptosis [5]. Initially implicated in pathologies such as neurodegenerative disorders, the role of ferroptosis in cancer has recently gained significant attention. Dysregulation of this pathway has been linked to both tumorigenesis and therapeutic resistance in various cancers, including melanoma [6]. This suggests that including ferroptosis could be a promising therapeutic approach for melanoma.

Ozonated vegetable oils, such as ozonated olive oil (OZO), are recognized for their therapeutic properties, including antimicrobial and wound-healing properties, leading to their use in cosmetics and medicine [[7], [8], [9]]. This activity is attributed to stable ozonides, primarily trioxolanes and peroxides, which are generated from the reaction between ozone and unsaturated fatty acids in vegetable oils [[7], [8], [9]]. In case of olive oil, its main component, triolein, is converted to triolein triozonide upon ozone treatment. Clinically, efficacy of OZO is well-established in wound-healing; for instance, it has been shown to accelerate healing of pressure ulcers in both mouse models and humans by suppressing suppuration and hemorrhage [10,11]. Although OZO's therapeutic effects on tissue repair are well-documented, its potential as an anti-cancer agent, particularly in melanoma where oxidative stress plays a key role, remains unexplored.

Thus, in this study, we evaluated anti-melanoma activity of OZO, focusing on its cytotoxic effects and therapeutic potential. We first evaluated the anti-proliferative efficacy and selectivity of OZO across a panel of human and murine melanoma cell lines in comparison to normal human keratinocytes. Subsequently, to uncover the underlying molecular basis of its action, we performed RNA-seq analysis to identify key signaling pathways modulated by OZO, which led us to establish ferroptosis as a primary hypothesis. Finally, we sought to directly validate this hypothesis by measuring key biochemical markers of ferroptosis such as lipid peroxidation and intracellular glutathione levels, and by utilizing specific cell death inhibitors to confirm that OZO-induced cell death is indeed mediated through this pathway.

## 2 Materials and methods

### 2.1 Reagents and antibodies

Ozonated olive oil (OZO; ORTHLY OIL®) and its precursor Olive oil (OLO) were purchased from Tamura Teco Co., Ltd (Osaka, Japan) and used as OZO without further purification. Caspase inhibitor, Z-VAD, and necrosis inhibitor, necrostatin-1 (Nec-1), were purchased from AdipoGen Life Science (San Diego, CA). Ferroptosis inhibitor, ferrostatin-1 (Fer-1), was purchased from Tokyo Chemical Industry (Tokyo, Japan). Iron chelator, deferiprone (DFP), was purchased from Cayman Chemical (Ann Arbor, MI). Lipid peroxide-specific reagent, liperfluo was purchased from DOJINDO (Kumamoto, Japan). α-tocopherol was purchased from FUJIFILM Wako Pure Chemical Corporation (Osaka, Japan). Anti-GAPDH Abs was purchased from Novus biological (Centennial, CO). Anti-GPX4 Abs was purchased from Proteintech (Rosemont, IL).

### 2.2 Cell culture

Human melanoma cell line, A375 cells, G361 cells and Malme-3M cells, human keratinocyte cell line, HaCaT cells and murine melanoma cell line, B16F10 cells were obtained from ATCC and cultured in Dulbecco's Modified Eagle Medium (DMEM; Sigma-Aldrich Co., St. Louis, MO) contained 10 % fetal bovine serum (FBS; Cosmo Bio Co., Tokyo, Japan) at 37 °C in humidified 5 % CO_2_/95 % air atmosphere.

### 2.3 Wound-healing assay

A375 cells were seeded into the wells of 24-well plates. 12 h after seeding, a scratch per well using a 200 μl pipette tip was made and a picture of the scratched area was taken. After that, the A375 cells were cultured with OLO or OZO-containing DMEM medium. 18 h later, a picture of the scratched area was again taken, and proportion of the uncovered area was measured using Image J (NIH, Bethesda, MD).

### 2.4 Proliferation assay

Cells were seeded on 96-well plates and were treated with the OLO and OZO. One or 2 days later, the cells were incubated with WST-8 (DOJINDO, Kumamoto, Japan) for 90 min at 37 °C and cell proliferation was monitored by micro plate reader.

### 2.5 Detection of lipid peroxides

Cells were cultured in 24-well plate. Twenty-four h after seeding, the cells were treated with 0.01 % OLO or OZO for 18 h. The cells were washed twice with Hank's Balanced Salt Solution (HBSS; FUJIFILM Wako Pure Chemical Corporation) and then incubated with 1 μM liperfluo-containing HBSS for 30 min at 37 °C. After that, cells were washed twice with HBSS, and fluorescence signal was observed by ZOE Fluorescent Cell Imager (Bio-Rad Laboratories, Inc., Hercules, CA). Lipid peroxidation was also monitored by flow cytometry, and mean fluorescence intensity was calculated.

### 2.6 Immunoblotting

Immunoblotting analysis was performed as described previously [12]. Densitometric analysis was performed using Image J software. Regions of interest (ROIs) corresponding to each band were delineated using the Rectangle tool. Background subtraction was performed in the plot lane by drawing a straight line across non-band regions with Straight line tool. Band intensities were then quantified by measuring the area under the valley profile using the Wand tool. Resulting values were normalized to the signal of the loading control protein for final quantification.

### 2.7 Quantitative PCR (qPCR) analysis

RNA isolation was performed using TRIzole reagent (Sigma-Aldrich) according to manufacturer's instructions. An equal amount of total RNA (1 μg) was used for generation of cDNAs using ReverTra Ace (TOYOBO, Osaka, Japan) according to manufacturer's instructions. qPCR analysis was performed using KAPA SYBR FAST qPCR Master Mix (NIPPON genetics Co, Ltd, Tokyo, Japan) for detection of gene expression. PCR primer sequences are shown in Table 1.

**Table 1:** Sequences of PCR primer sets.

*ACTB*	Sense	CCAACCGCGAGAAGATGA
*ACTB*	Antisense	CCAGAGGCGTACAGGGATAG
*FTH1*	Sense	GAGTGACAAGCCTGTAGCCCATGTTGTAGC
*FTH1*	Antisense	GGCAATGATGATCCCAAAGTAGACCTGCCC
*FTL*	Sense	ACTACCACCAGGACTCAGAGG
*FTL*	Antisense	AGTCATCACAGTCTGGTTTCTTGA
*GCLM*	Sense	CGCACAGCGAGGAGGAGTTT
*GCLM*	Antisense	AATCCAGCTGTGCAACTCCAA
*GCLC*	Sense	ACTGCTCACCAGAGTGATCC
*GCLC*	Antisense	CCACTGCATTGCCACCTTTG
*HMOX1*	Sense	GCTCTGGTCCTTGGTGTCAT
*HMOX1*	Antisense	CTTCTTCACCTTCCCCAACA
*SLC7A11*	Sense	TGTGTGGGGTCCTGTCACTA
*SLC7A11*	Antisense	CAGTAGCTGCAGGGCGTATT
*GPX4*	Sense	AGAGATCAAAGAGTTCGCCGC
*GPX4*	Antisense	TCTTCATCCACTTCCACAGCG
*SQSTM1*	Sense	GAGATTCGCCGCTTCAGCTT
*SQSTM1*	Antisense	GGAAAAGGCAACCAAGTCCC
*Actb*	Sense	TGACAGGATGCAGAAGGAGA
*Actb*	Antisense	CGCTCAGGAGGAGCAATG
*Hmox1*	Sense	TTGGTGGCCTCCTTCAAGG
*Hmox1*	Antisense	GTGATGGAGCGTCCACAGC
*Gclm*	Sense	CCCCGATTTAGTCAGGGAGT
*Gclm*	Antisense	TCTTCAATCGGAGGCGAAGC
*Gclc*	Sense	AAGGACGTGCTCAAGTGGG
*Gclc*	Antisense	CAGAGGGTCGGATGGTTGGG
*Fth1*	Sense	CATCAACCGCCAGATCAACC
*Fth1*	Antisense	GTCATCACGGTCTGGTTTCTTTAT
*Ftl1*	Sense	GCGGACCCTCATCTCTGTGA
*Ftl1*	Antisense	GAGCCCCTTGGAAGGTACAG
*Sqstm1*	Sense	GGGACAGCCAGAGGAACAGA
*Sqstm1*	Antisense	GGAGAGGGACTCAATCAGCC

### 2.8 RNA-sequencing (RNA-seq) library construction, library sequencing and data analysis

Total RNA was extracted from A375 and B16F10 cells by using TRIzol reagent. Library preparation, sequencing and FASTQ file generation were outsourced to Azenta Japan Corp. (Tokyo, Japan). Libraries were generated using the NEBNext Ultra II Directional RNA Library Prep Kit (New England Biolabs). All libraries were sequenced (2 × 150 bp) using Illumina NovaSeq 6000 (Illumina, San Diego, CA, USA). FASTQ files were aligned to the reference human genome (hg38) or reference mouse genome (mm10) using HISAT2 (version 2.1.0). The StringTie algorithm (v.1.3.4d) was then used to assemble RNA-Seq alignments into annotated transcripts and estimate their expression using the UCSC annotated human genome (hg38) or mouse genome (mm10) assembly file. Subsequently, the transcript expression was normalized using the transcripts per million (TPM) algorithm. For differential expression analysis, we used feature Counts (v2.1.1+galaxy0) and limma-voom (v3.58.1+galaxy0).

### 2.9 Intracellular GSH/GSSG measurement

Intracellular GSH/GSSG ratio was measured by using GSH/GSSG quantification kit (DOJINDO) according to the manufacturer's protocol. Cells were seeded on 6 cm dishes one day before treatment. After treatment with OLO or OZO, the cells were collected by trypsinization. Intracellular total glutathione isolation of cell pellets was performed by using 1 % sulfosalicylic acid (SSA)/PBS solution.

### 2.10 Statistical analysis

Statistical analysis was performed by using GraphPad prism (v6.0.2). To determine statical significance between two groups, we used Welch's *t*-test. To determine statical significance between three or more groups, One-way analysis of variance (ANOVA) followed by Tukey's multiple-comparisons test was used. Data are presented as mean + or ± SEM, and differences were considered significant at p < 0.05.

## 3 Results

### 3.1 OZO inhibits cell migration and proliferation in melanoma cells

**Fig. 1:**
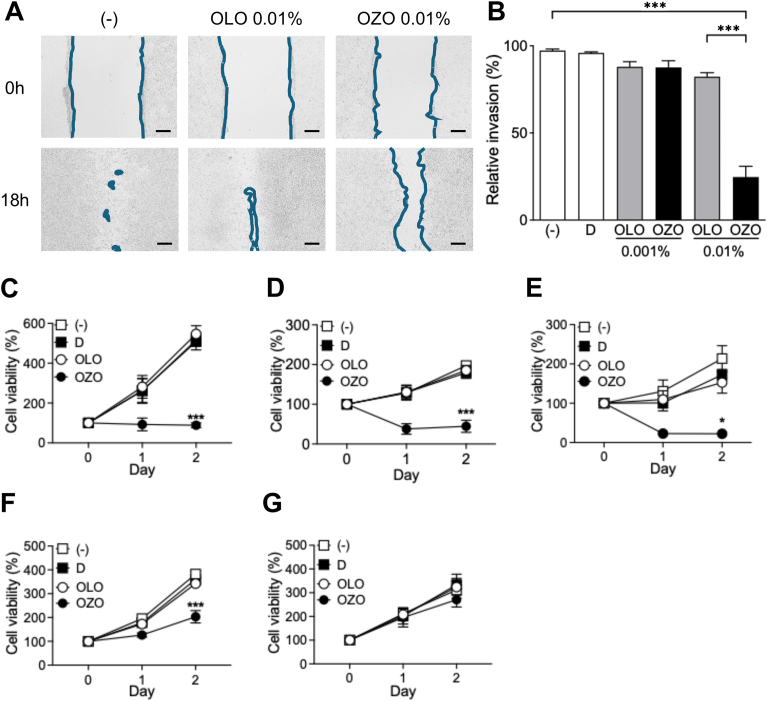
Ozonated olive oil has ability to inhibit cell migration and proliferation in melanoma cells (A) Seeded A375 cells were scratched (top panels) and incubated with or without 0.001 % or 0.01 % OZO or OLO for 18 h (bottom panels). Blue lines are shown as borders of seeded cells. The images are representative of 3 independent experiments (n = 3). Scale bars, 200 μm. (B) Proportion of invasion areas in OZO-treated and OLO-treated A375 cells are shown. Data were shown mean + SEM of 3 independent experiments. (C–G) Cell viability of A375 (C), G361 (D), Malme-3M (E), B16F10 (F) and HaCaT (G) cells in the presence of 0.01 % OZO (filled circles) or OLO (open circles). Data were shown mean + SEM of 3 independent experiments (n = 3). Statistical analysis performed by Tukey's multiple comparison test. ∗; p < 0.05, ∗∗∗; p < 0.001 (vs OLO). (−): no addition, D: DMSO-treated.

To evaluate the effect of OZO on melanoma cell migration, we conducted an *in vitro* wound-healing assay. Confluent A375 cell were scratched and then treated with OLO or OZO, or vehicle control (DMSO). Control groups (untreated (−) and DMSO-treated) and OLO-treated A375 cells showed nearly complete wound closure (Fig. 1A, left and middle panels). In contrast, OZO treatment significantly inhibited the migration of A375 cells, as shown by the larger remaining scratch area (Fig. 1A, right panels, and 1B).

Next, we investigated whether OZO also inhibits the proliferation of human and murine melanoma cells. Treatment with 0.01 % OZO significantly suppressed the proliferation of multiple human (A375, G361, Malme-3M) and murine (B16F10) melanoma cell lines, compared to 0.01 % OLO-treatment (Fig. 1C–F). To determine if this effect was specific to cancer cells, we examined that the same assay on normal human keratinocytes (HaCaT) cell line. Notably, OZO did not affect the proliferation of HaCaT cells (Fig. 1G). Taken together, these results indicate that OZO suppresses the migration and proliferation of melanoma cells.

### 3.2 OZO induces a ferroptotic gene expression in melanoma cells

**Fig. 2:**
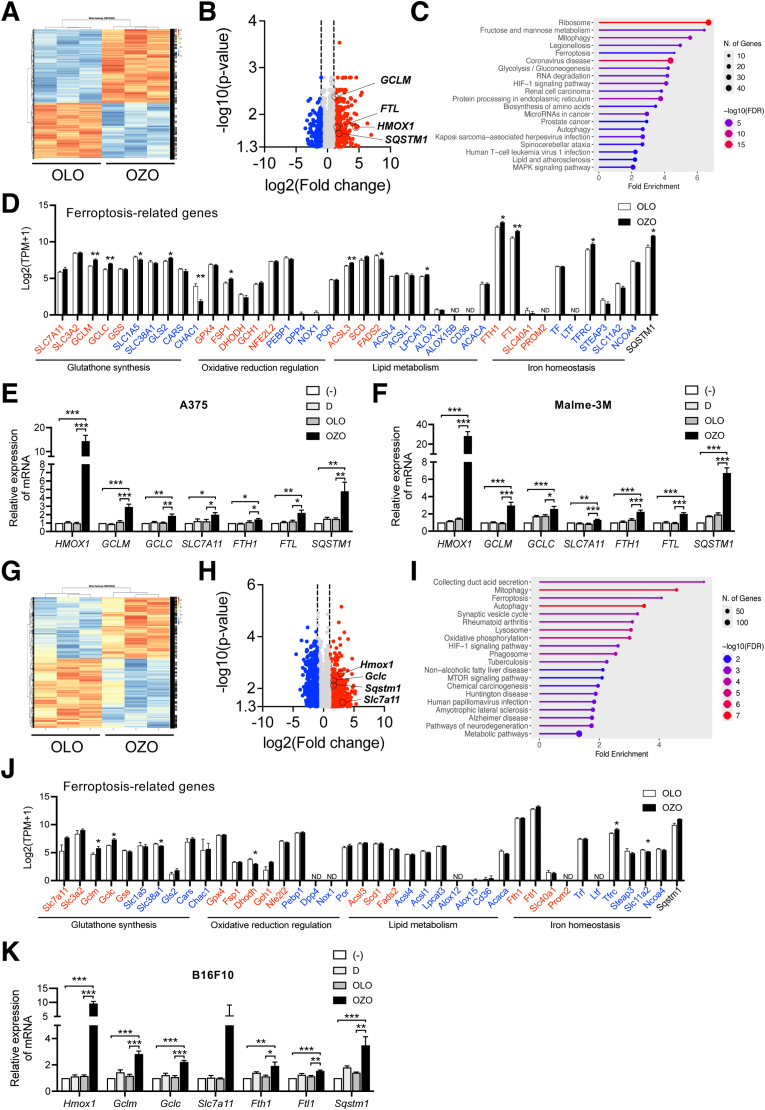
Gene expression analysis in human and murine melanoma cells (A-C) Heatmap image (A) Volcano plot (B) and KEGG pathway enrichment analysis (C) of A375 cells in the presence of 0.01 % OZO or OLO (n = 3) by comprehensive gene expression analysis. (D) Transcriptomic analysis of ferroptosis-related gene mRNA expression changes in A375 cells Data were shown as mean + SEM of 3 independent experiments (n = 3). Statistical analysis was performed by Welch's *t*-test. Red: ferroptosis inhibition-related genes, Blue: ferroptosis induction-related genes. (E, F) Expression of ferroptosis-related gene in A375 (E) and Malme-3M (F) cells at 6 h after treatment with 0.01 % OZO or 0.01 % OLO. Data were shown mean + SEM of 4 independent experiments (n = 4). Statistical analysis was performed by Tukey's multiple comparison test. (G–I) Heatmap image (G) Volcano plot (H) and KEGG pathway enrichment analysis (I) of B16F10 cells in the presence of 0.01 % OZO or OLO (n = 3) by comprehensive gene expression analysis. (J) transcriptomic analysis of ferroptosis-related gene expression in B16F10 cells Data were shown as mean + SEM of 3 independent experiments (n = 3). Statistical analysis was performed by Welch's *t*-test. Red: ferroptosis inhibition-related genes, Blue: ferroptosis induction-related genes. (K) Expression of ferroptosis-related genes in B16F10 cells at 6h after treatment with 0.01 % OZO or 0.01 % OLO. Data were shown mean + SEM of 4 independent experiments (n = 4). Statistical significance by Tukey's multiple comparison test. ∗; p < 0.05, ∗∗; p < 0.01, ∗∗∗; p < 0.001. (−): no addition, D: DMSO-treated.

To elucidate the molecular mechanism underlying the anti-proliferative effect of OZO, we first conducted a comprehensive transcriptomic analysis using RNA-sequencing (RNA-seq) on human melanoma A375 cells. This unbiased approach reveals a substantial shift in the gene expression landscape, identifying 1984 differentially expressed genes (DEGs), with 1121 upregulated and 863 downregulated in OZO-treated A375 cells compared to the OLO control (Fig. 2A and B). To understand the biological significance of these changes, we performed pathway enrichment analysis. Notably, KEGG [13] pathway analysis pointed to ferroptosis as one of the most significantly enriched pathways among the DEGs (Fig. 2C), suggesting its potential role in OZO's mechanism of action. A closer inspection of genes within this pathway highlighted the significant upregulation of key ferroptosis regulator, including *GCLM, HMOX1, FTL*, and *SQSTM1* (Fig. 2D). We validated these critical findings via RT-qPCR, which confirmed a robust increase in the mRNA levels of these markers in A375 cells (Fig. 2E). This ferroptotic response was not limited to a single cell line, as a similar upregulation pattern was reproduced in another human melanoma cell line, Malme-3M (Fig. 2F).

Building on these findings, we next sought to investigate whether this ferroptotic mechanism by OZO is conserved across species. To this end, we performed a parallel RNA-seq analysis on the murine melanoma cell line, B16F10. The transcriptome of OZO-treated B16F10 cells also underwent extensive reprogramming, with 3557 DEGs identified (1665 upregulated, 1892 downregulated) (Fig. 2G and H). Corroborating our observations in human melanoma cells, KEGG pathway enrichment analysis of upregulated DEGs in the murine cells again revealed a significant enrichment for the ferroptosis pathway (Fig. 2I). This conservation was evident at the individual gene level, with key ferroptosis-regulating genes such as *Hmox1*, *Gclc*, *Slc7a11 and Sqstm1* being markedly upregulated (Fig. 2J). Subsequent RT-qPCR analysis provided further validation, confirming a significant increase in the expression of ferroptosis-related genes, including *Hmox1, Gclm, Gclc, Fth1, Ftl1 and Sqstm1* (Fig. 2K). Taken together, these results demonstrate that OZO induces expression of ferroptosis-related genes in both human and murine melanoma cells, indicating a conserved mechanism of action.

### 3.3 OZO treatment elicits the canonical biochemical hallmarks of ferroptosis

**Fig. 3:**
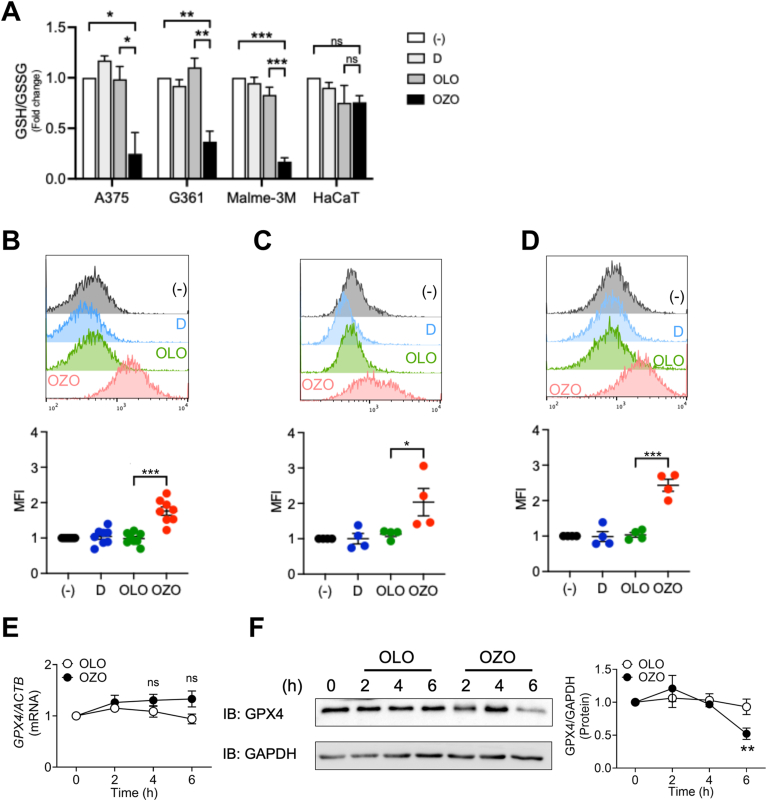
Ozonated olive oil induces ferroptosis in melanoma cells (A) Level of intracellular GSH and GSSG ratio in A375, G361, Malme-3M and HaCaT cells treated with or without 0.01 % OLO or 0.01 % OZO for 6h. Data was shown mean + SEM of 3 independent experiments (n = 3). (B–D) Levels of lipid peroxidation in A375 (B), G361 (C) and Malme-3M (D) cells treated with or without 0.01 % OZO- or 0.01 % OLO analyzed by flowcytometric analysis. (Upper panel) The images are representative of individual independent experiments. (Lower panel) The graphs are shown that measured Geometric MFI from 8 (A375), and 4 (other) independent experiments. Statistical significance by Tukey's multiple comparison test. ∗; p < 0.05, ∗∗∗; p < 0.001. (−): no addition, D: DMSO-treated. (E–F) Expression of GPX4 mRNA and protein in A375 at 0–6h after treatment with 0.01 % OLO or 0.01 % OZO. (E) The graph is shown that fold change of GPX4 mRNA levels. Data was shown mean + SEM of 5 independent experiments (n = 5). (F). Data are representative of 5 independent experiments. (F, right panel) The graph is shown that measured GPX4 and GAPDH protein ratio by Image J from 5 independent experiments (n = 5). Statistical significance by Tukey's multiple comparison test. ns; not significant, ∗∗; p < 0.01 (vs OLO).

Having established that OZO induces a ferroptotic gene signature, we next investigated whether this translates to the core biochemical event of ferroptosis. This process is initiated by the depletion of intracellular glutathione (GSH), which cripples the cell's antioxidant defenses and leads to the accumulation of lipid peroxides [14]. Accordingly, we found that OZO treatment caused a significant depletion of GSH in melanoma cells, an effect not observed in non-cancerous HaCaT keratinocytes, highlighting a cancer-cell specific action (Fig. 3A). Consistent with GSH depletion, OZO also triggered a significant increase in lipid peroxidation, a key executioner of ferroptotic cell death, across multiple melanoma cell lines, including A375, G361 and Malme-3M (Fig. 3B–D).

Given that the selenoenzyme Glutathione peroxidase 4 (GPX4) is a key regulator of lipid hydroperoxide detoxification [[15], [16], [17]], we next examined its status. While OZO treatment did not affect GPX4 mRNA levels (Fig. 3E), Western blot analysis revealed a marked reduction in GPX4 protein levels in A375 cells (Fig. 3F). This discrepancy suggests a post-transcriptional mechanism of GPX4 downregulation. Taken together, these results demonstrate that OZO triggers ferroptosis through a dual mechanism: depleting the substrate GSH and downregulating the key enzyme GPX4. This two-pronged attack effectively dismantles the cell's primary defense against lipid peroxidation, leading to its lethal accumulation.

### 3.4 OZO-induced cell death is rescued by ferroptosis inhibitors and modulated by iron

**Fig. 4:**
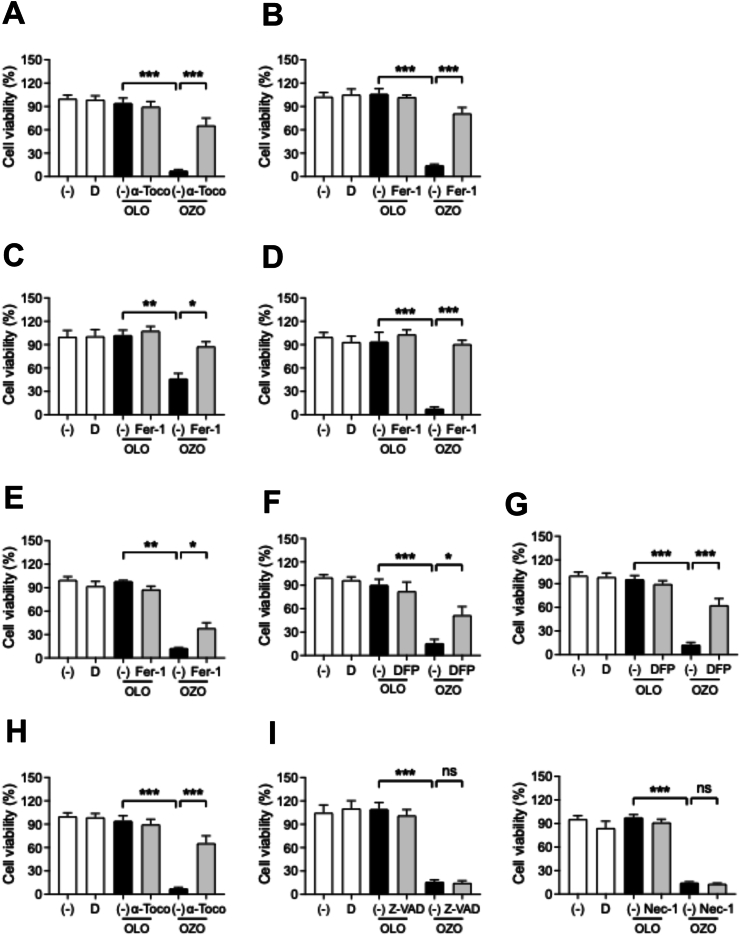
Ferroptosis inhibitors but not apoptosis and necroptosis inhibitor suppressed ozonated olive oil-induced melanoma cell death (A-B) Cell viability of 0.01 % OZO- or 0.01 % OLO-treated A375 cells in the presence or absence of 10 μM α-tocopherol (α-Toco) (A), 1 μM ferrostatin-1 (Fer-1) (B) Data was shown mean + SEM 4 of individual independent experiments (n = 4). ∗∗∗; p < 0.001 by Tukey's multiple comparison test. (C–E) Cell viability of 0.01 % OZO- or 0.01 % OLO-treated G361 (C), Malme-3M (D) and B16F10 (E) cells in the presence or absence of ferrostatin-1 (Fer-1). (F, G) Proliferation of 0.01 % OZO- or OLO-treated A375 (F) and Malme-3M (G) cells in the absence or presence of 100 μM deferiprone (DFP). Data was shown mean + SEM of 4 individual independent experiments (n = 4). (H) Proliferation of 0.003 % OZO- or OLO-treated A375 cell in the absence or presence of 100 μM FeSO_4_. Data was shown mean + SEM of 3 individual independent experiments (n = 3). (I) Proliferation of 0.01 % OZO- or OLO-treated A375 cells in the absence or presence of 10 μM Z-VAD (left panel) or 1 μM necrostatin-1 (Nec-1, right panel). Data was shown mean + SEM of 4 individual independent experiments (n = 4). Statistical significance by Tukey's multiple comparison test. ns; not significant, ∗; p < 0.05, ∗∗; p < 0.01, ∗∗∗; p < 0.001. (−): no addition, D: DMSO-treated.

Our preceding data showed that OZO induces multiple hallmarks of ferroptosis in melanoma cells (Fig. 2, Fig. 3). To confirm that ferroptosis is the primary mechanism of melanoma cell death, we performed rescue experiments using established ferroptosis inhibitors [5,18]. Co-treatment with the lipid ROS scavengers ferrostatin-1 (Fer-1) or α-tocopherol (α-Toco) significantly reversed the anti-proliferative effect of OZO in A375 cells (Fig. 4A–B). This rescue effect was consistently observed with Fer-1 in other human and murine melanoma cell lines (Fig. 4C–E).

A defining characteristic of ferroptosis is its strict dependence on intracellular labile iron [19,20]. We therefore sought to confirm this by manipulating iron availability. Pre-treatment with the iron chelator deferiprone (DFP) effectively sequestered intracellular iron and, as a result conferred significant protection against OZO-induced cell death in both A375 and Malme-3M cells (Fig. 4F and G). Conversely, supplementing cells with ferrous sulfate (FeSO_4_) to increase the labile iron pool dramatically sensitized them to OZO, potentiating cell death even at a previously non-toxic concentration (Fig. 4H). These opposing outcomes from iron chelation and supplementation provide unequivocal evidence that OZO's cytotoxicity is directly modulated by iron-dependent Fenton reaction.

Finally, to establish the specificity of the ferroptotic pathway, we investigated the potential involvement of other programmed cell death mechanisms. Co-treatment with pan-caspase inhibitor Z-VAD (apoptosis inhibitor) or the necroptosis inhibitor necrostatin-1failed to provide any protective effect against OZO-induced lethality (Fig. 4I). Taken together, these findings provide compelling evidence that OZO specifically triggers iron-dependent ferroptosis to suppress melanoma cell survival.

## 4 Discussion

In this study, we elucidate a novel mechanism for the anti-melanoma activity of OZO, demonstrating that it suppresses cell proliferation by inducing ferroptosis. This conclusion is supported by a comprehensive set of evidence: OZO treatment not only altered the expression of key ferroptosis-related genes but also caused a significant accumulation of lipid peroxidation. Crucially, the cytotoxic effects of OZO were completely rescued by treatment with specific ferroptosis inhibitors. Taken together, our findings establish OZO as a potent inducer of ferroptosis and highlight its potential as a promising therapeutic agent for melanoma.

Targeting the ferroptosis has emerged as a promising therapeutic strategy in oncology. For instance, the canonical ferroptosis inducer (FIN), Erastin, has been shown to deplete GSH, increased lipid peroxidation, and suppresses tumor growth in a mouse model of diffuse large B cell lymphoma [21]. Furthermore, the clinically approved multi-kinase inhibitor sorafenib, used for advanced hepatocellular carcinoma, exerts its anti-cancer effects partly by triggering ferroptosis-mediated tumor cell death. Notably, resistant to sorafenib has been associated with the downregulation of SLC27A5, a gene crucial for fatty acid metabolism whose deficiency impairs the execution of ferroptosis [22,23].

A major clinical challenge in melanoma metastasis, which is the primary driver of patient mortality [24]. It is well-established that metastatic melanoma cells endure heightened oxidative stress. To survive this hostile environment, they become heavily reliant on antioxidant defense system, such as to enhance NADPH production and GSH regeneration to maintain redox homeostasis [25,26]. This dependency creates a key therapeutic vulnerability, making them particularly susceptible to agents that disrupt these defenses [27]. Our findings align with this concept. In this study, we have demonstrated that OZO potently triggers ferroptosis by disrupting GSH-GPX4 axis, the very system that metastatic cells rely on for survival. These findings suggest that OZO holds significant promise not only for treating primary tumours but also as a potent agent for inhibiting melanoma metastasis.

While GPX4 mRNA level remained unchanged, we observed a statistically significant decrease in GPX4 protein levels, suggesting the involvement of prost-transcriptional regulatory mechanisms. Recent studies have reported that autophagy can mediate GPX4 degradation and thereby promote ferroptosis [28]. Given that OZO is a complex oxidizing agent, it is plausible that OZO might trigger an autophagy-dependent pathway leading to GPX4 degradation. Therefore, future study investigating the interplay between OZO-induced autophagy and GPX4 protein stability are essential to precisely delineate the molecular mechanisms driving ferroptosis in melanoma cells.

Combining ferroptosis induction with established cancer therapies like immunotherapy and chemotherapy has recently been demonstrated [29]. In melanoma, long-term BRAF inhibitor treatment or acquired resistance increased sensitivity to ferroptosis, as seen with erastin and RSL3 treatments [30]. Additionally, inducing ferroptosis by depleting cysteine, methionine restriction, or targeting SLC7A11 and GPX4 enhances immunotherapy efficacy in xenograft mouse models [4,21,31]. In this context, our study demonstrates that OZO treatment induces a activation of the NRF2 antioxidant pathway. This is consistent with the action of other ferroptosis inducers, suggesting it represents a general adaptive response of melanoma cells to the inherent oxidative stress of ferroptosis. While this NRF2 activation provides a pro-survival signal, it is ultimately insufficient to counteract the potent pro-ferroptotic effect of OZO. Combining OZO with an NRF2 inhibitor could represent a rational strategy to synergistically enhance its anti-tumour efficacy by dismantling the cell's primary defense system against oxidative insults.

While this study demonstrates the potent ferroptosis-inducing activity of OZO, a key limitation is that OZO is a complex mixture, and the specific active component has not yet been identified. Based on the chemical nature of OZO, we hypothesize that the ozonide functional group is the primary driver of this effect likely by initiating lipid peroxide. Therefore, a crucial next step is to validate this hypothesis by determining if a purified, single-component lipid ozonide [32] can recapitulate the anti-melanoma effects observed with OZO. Such studies would also allow for a more detailed mechanistic exploration, providing a strong rationale for combining this targeted approach with existing therapies, like immunotherapy, to enhance treatment outcomes for melanoma patients.

## CRediT authorship contribution statement

**Seong-Jin An:** Writing – original draft, Visualization, Investigation. **Jun-Ichi Kashiwakura:** Writing – review & editing, Writing – original draft, Visualization, Investigation. **Akira Katsuyama:** Investigation. **Sumihito Togi:** Investigation. **Yuichi Kitai:** Supervision. **Ryuta Muromoto:** Supervision. **Toshiaki Miura:** Writing – review & editing, Writing – original draft, Supervision. **Satoshi Ichikawa:** Supervision. **Tadashi Matsuda:** Writing – review & editing, Writing – original draft, Visualization, Supervision.

## Declaration of competing interest

The authors declare that they have no known competing financial interests or personal relationships that could have appeared to influence the work reported in this paper.

## Figures and Tables

**Fig. S1:**
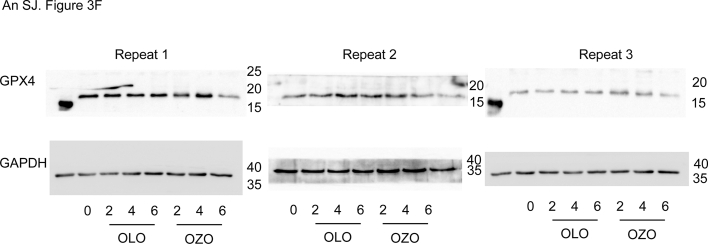


## Data Availability

The microarray data presented in this article have been submitted to the Gene Expression Omnibus under accession number GSE306902 and GSE306903. The other data supporting the findings of this study are available within the paper or can be obtained from the corresponding author upon reasonable request.
